# Outcomes of Hypertensive Kidney Donors Using Current and Past Hypertension Definitions

**DOI:** 10.1016/j.ekir.2021.02.034

**Published:** 2021-03-03

**Authors:** Hassan N. Ibrahim, Sean A. Hebert, Dina N. Murad, Horacio E. Adrogue, Duc T. Nguyen, Edward A. Graviss, Hana Nguyen, Arthur Matas

**Affiliations:** 1 Division of Renal Diseases, Hypertension and Transplantation, Houston Methodist Hospital, Houston, Texas, USA; 2 Department of Pathology and Genomic Medicine, Institute for Academic Medicine, Houston Methodist Hospital, Houston, Texas, USA; 3 Department of Surgery, Houston Methodist Hospital, Houston, Texas, USA; 4 Department of Surgery, University of Minnesota, Minneapolis, Minnesota, USA

**Keywords:** donor, hypertension, kidney

## Abstract

**Introduction:**

As many as 50% of U.S. transplant centers do not accept kidney donor candidates with hypertension, citing the link between hypertension, kidney disease, and cardiovascular disease (CVD).

**Methods:**

We ascertained mortality, CVD, proteinuria, estimated glomerular filtration rate (eGFR) trajectory, reduced eGFR, and end-stage kidney disease (ESKD) in 904 hypertensive donors (blood pressure [BP] ≥140/90 mm Hg or receiving treatment) versus 7817 donors with BP <140/90 mm Hg.

**Results:**

Hypertensive donors were older, 58.1% were <50 years of age, and they had a lower eGFR. The majority were white and related to their recipient. At the end of follow-up, 14.3 ± 10.1 years (range 4–48 years) from donation, hypertensive and nonhypertensive donors had a similar prevalence of cardiovascular disease and renal outcomes. The multivariable risk of mortality, CVD, and proteinuria were also comparable in normotensive and hypertensive donors. eGFR slope over time was similar in hypertensive and nonhypertensive donors, and in total 5 hypertensive and 39 normotensive donors developed ESKD 19.2 ± 10.3 years after donation (adjusted hazard ratio 1.14 [95% confidence interval 0.62–2.12], *P* = 0.67). Sensitivity analysis using the new definition of hypertension (≥130/80 mm Hg or requiring treatment) yielded similar results for renal outcomes, but hypertensive donors were more likely to develop CVD and diabetes.

**Conclusions:**

Kidney donors with hypertension defined by past criteria do not appear to incur higher mortality, CVD, or ESKD. Donors with current definition of hypertension enjoyed similar renal outcomes but were more likely to develop CVD.

## Graphical abstract

**Figure:**
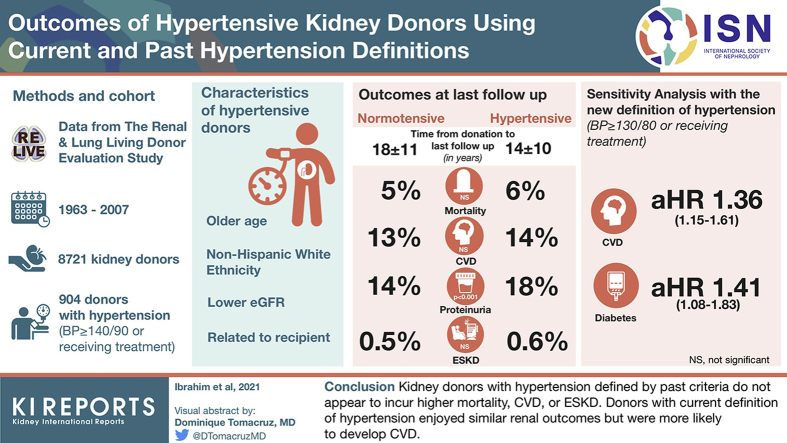

See Commentary on Page 1208

Reductions in renal mass and function are associated with an increase in blood pressure and the development of systemic hypertension in animal models and humans with reduced renal mass.^1^^,^^2^ Hypertension is widely cited as the second leading cause of ESKD in the United States, and ESKD in many former kidney donors has been attributed to hypertension.^3^^,^^4^ Moreover, a recent analysis suggests that roughly a third of kidney donors developed hypertension 15 years after donation compared with <10% in nondonor healthy control subjects, and in a separate analysis from the same investigators, predonation hypertension in donors >50 years of age was associated with an overall ESKD incidence of <1%.^5^^,^^6^ The kidney- and cardiovascular-related concerns regarding hypertensive candidates are reflected by exclusions and also restrictions put on donor candidates with hypertension by many transplant centers.^7^

Hypertension accelerates the progression of established kidney disease, but the strength of the causal link between hypertension and incident chronic kidney disease (CKD) is not definitive. In many cases, hypertension follows the development of CKD rather than precedes it.^8^ In addition, many patients with advanced CKD who are labeled as having hypertensive nephrosclerosis not infrequently have focal segmental glomerulosclerosis and other glomerular pathologies on review of a kidney biopsy specimen.^8^^,^^9^ Moreover, strict BP control in the SPRINT study, while hugely associated with lower mortality, did not appear to lower CKD incidence, at least by its classic definition of eGFR <60 ml/min/1.73 m^2^.^10^

The definition of hypertension has evolved over the years, and therefore many kidney donors who donated in the past, particularly early on in transplantation history, would be considered hypertensive by today’s standards. This provides a unique opportunity to study the impact of isolated hypertension on long-term kidney function because donors have no evidence of any renal involvement, such as proteinuria or low GFR, and no major comorbidities at donation, and therefore the temporal relationship between hypertension and kidney disease can be better dissected. Moreover, such knowledge may also inform our current selection criteria pertaining to donor candidates with hypertension. Lastly, determining outcomes of donors using the newly introduced hypertension definition (≥130/80 mm Hg) would shed light on the size of the overall kidney donor pool if centers were to use it for donor eligibility.^11^

## Methods

We used publicly available data from The Renal and Lung Living Donor Evaluation (RELIVE) study, a National Institute of Allergy and Infectious Diseases (NIAID)–sponsored study that evaluated outcomes of 8922 kidney donors from 3 U.S. transplant centers: the University of Minnesota, Mayo Clinic-Rochester, and the University of Alabama-Birmingham. All donations took place between 1963 and 2007. Donors’ medical records were abstracted at each of the participating centers for baseline information, which included demographic information, anthropometric measurements, previous or current diagnosis or treatment for hypertension or hyperlipidemia, and laboratory data, as previously described.^12^ Family history of hypertension, diabetes mellitus, kidney disease, stroke, or heart disease were also recorded.

BP readings were collected on multiple occasions during the donor evaluation, and the average of the 3 lowest readings was used as baseline to minimize misclassifying donors with white coat hypertension as truly hypertensive as described by Taler *et al.*^12^ Hypertension was defined by the extant definition at the time of the study, which was BP ≥140/90 mm Hg or the requirement for antihypertensive agents. Between 2010 and 2012, the 3 RELIVE study centers contacted donors by mail requesting participation in the RELIVE study. If no response was received, a follow-up letter and ≥2 phone calls were made by study personnel. In addition, a fee-based internet service was used to update donors’ addresses and phone numbers (Accurinet; www.accurint.com). Donors were asked to provide responses to quality of life surveys and were asked about developing diabetes, hypertension, kidney disease, CVD, cancer, and other conditions. In addition, participating centers provided all follow-up data they had on their own donors. In many instances, recipients also provided information about their donors. Postdonation diabetes mellitus was considered present if it was self-reported by the donor, a fasting plasma glucose ≥126 mg/dl from laboratory work conducted any time after donation, the requirement for insulin, oral hypoglycemic agents, or evidence of end organ damage (retinopathy or nephropathy). Postdonation hypertension was defined as use of antihypertensive medications specifically used for hypertension treatment or a documented home, center, or office-based BP ≥140/90 mm Hg. CVD was defined as a diagnosis of myocardial infarction, congestive heart failure, stroke, or the need for coronary or peripheral arterial interventions. Proteinuria was defined as any of the following: urine dipstick protein ≥2+, urine protein/osmolality ratio >0.42, urine random protein >15 mg/dl, or 24-hour protein >300 mg/day. The Chronic Kidney Disease Epidemiology Collaboration equation was used to calculate the eGFR.^13^ ESKD was defined by the need for dialysis or being listed for or receiving a transplant. The ascertainment of ESKD in this public dataset was from centers’ records, donors themselves, or their recipients.

### Statistical Analysis

Demographic and clinical data are reported as frequencies and proportions for categorical variables and as median and 25th to 75th percentile for continuous variables. Differences between hypertensive and nonhypertensive donors were compared using the Pearson χ^2^ or Fisher exact tests for categorical variables and the Kruskal Wallis test for continuous variables. Two analyses were conducted, first according to hypertension status defined by BP ≥140/90 mm Hg or requirement for antihypertensive agents and the second according to the newer definition of normotension as BP <130/80 mm Hg versus ≥130/80 mm Hg or requiring treatment. Donor age, fasting plasma glucose, and body mass index (BMI) were evaluated as both continuous and categorical variables. Cox regression modeling was conducted for the following postdonation outcomes: death, death-censored diabetes, hypertension, proteinuria, eGFR <60, <45, and <30 ml/min/1.73 m^2^, CVD, ESKD, and a composite of eGFR <30 ml/min/1.73 m^2^ or ESKD. The latter was used to detect earlier degree of renal dysfunction as ESKD alone is a rare event after donation. The selection of variables for the initial Cox proportional hazard models were conducted using the Stata Lasso command with the cross-validation selection method and also by the clinical importance and biologic plausibility as determined by the authors.^14^^,^^15^ Cox proportional hazard models for outcomes other than postdonation hypertension included systolic BP (SBP) at evaluation, donor age, gender, BMI, fasting plasma glucose, eGFR at evaluation, smoking, hyperlipidemia, and 2 time-varying variables (development of hypertension and diabetes). Baseline SBP and eGFR were not included in the models for postdonation hypertension. For eGFR <30 ml/min/1.73 m^2^, ESKD, and eGFR <30 ml/min/1.73 m^2^ or ESKD outcomes, only age, gender, BMI at evaluation, and eGFR at evaluation were included in the models given the small number of events. Model discrimination was assessed using the Harrell C-statistic.^16^ The proportional hazards assumption was also evaluated for the Cox proportional hazards models. Outcomes other than death were censored for death. Cumulative incidence for outcomes other than death was estimated using the competing risk method described by Fine and Gray.^17^ Multiple imputation by chained equations was used to impute missing baseline data for fasting plasma glucose (5.6% missing), eGFR (0.4% missing), relation to the recipients (0.3% missing), smoking (2.5% missing), and hyperlipidemia (0.7% missing). The trend of eGFR over time was constructed using the median cubic spline plots. The difference in the change of eGFR over time between the hypertensive and nonhypertensive donors was compared using the linear mixed model.

All analyses were performed using Stata software (version 16.1; StataCorp LLC, College Station, TX, USA). *P* < 0.05 was considered statistically significant.

## Results

### General Characteristics of RELIVE Study Donors

**Figure 1:**
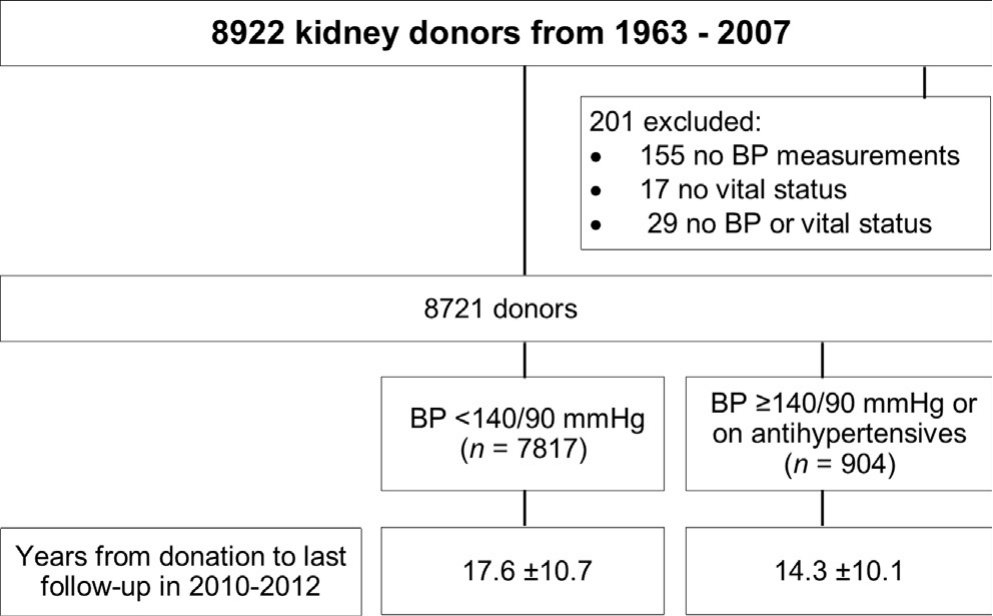
Study participants. BP, blood pressure.

**Figure 2:**
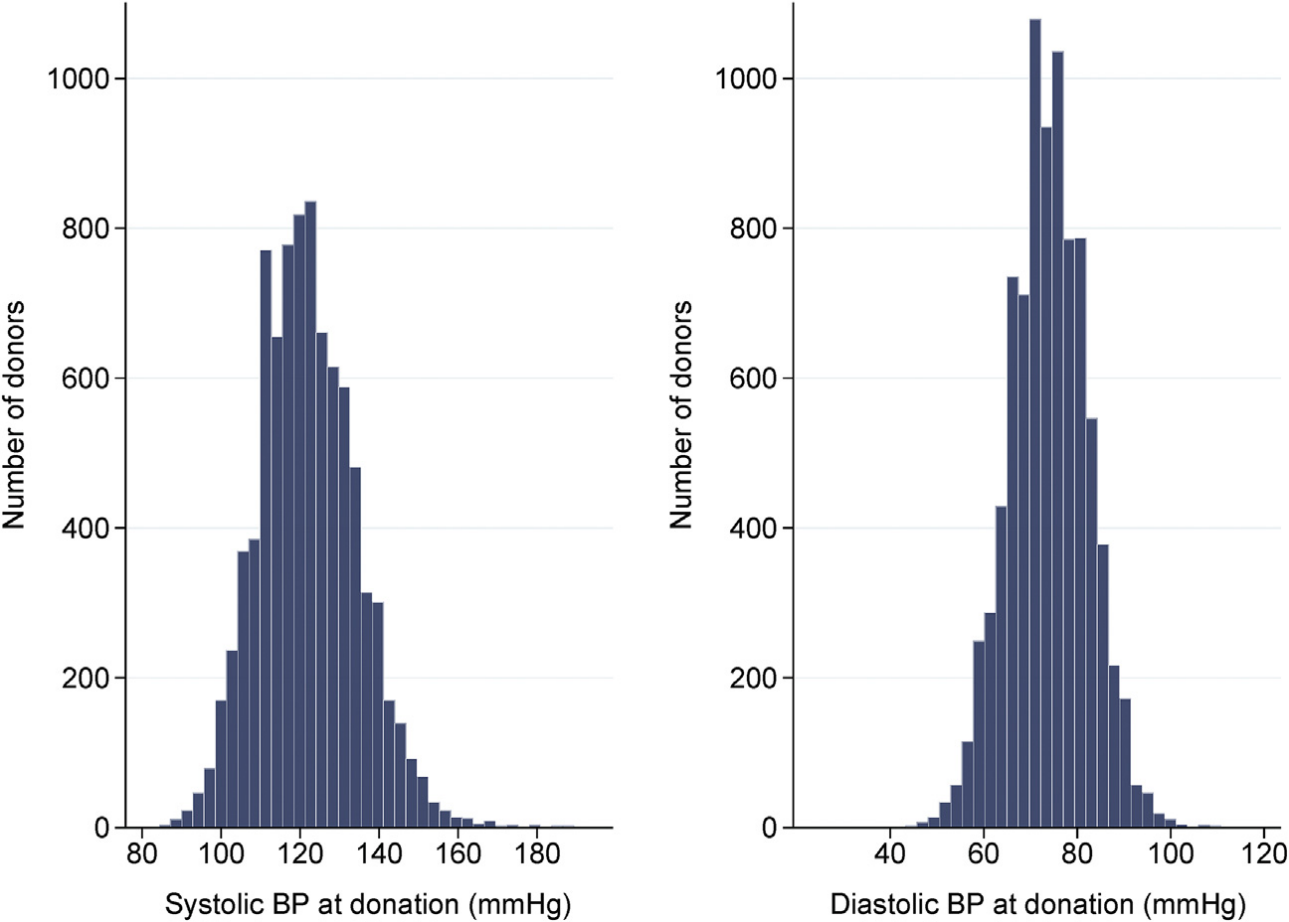
Blood pressure (BP) distribution at donation.

Of 8922 kidney donors, 8721 donated a kidney between 1963 and 2007, had multiple predonation BP measurements available, and had their vital statuses ascertained (Figure 1). Vital status was ascertainable in 99.8% of the donors, CVD in 98%, eGFR value in 97.1%, postdonation hypertension in 98%, postdonation diabetes in 90.2%, and proteinuria data in 89.9%. The median age at donation of the entire cohort was 39 years, 56.2% were women, 85% were non-Hispanic white, 9.2% were non-Hispanic black, 1.8% were Hispanic, 0.9% were Asian, and 3% were categorized as other. The majority (80.5%) donated to a family member; 71% had ≥1 first-degree relative with kidney disease and 41% with ≥1 first-degree relative with hypertension. The median BMI was 25.8 kg/m^2^ and the median eGFR was 88 ml/min/1.73 m^2^. In total, 6352 (72.8%) had SBP <130 mm Hg, 1465 (16.8%) had 130 ≤ SBP < 140 mm Hg, 653 (7.5%) had SBP ≥140 mm Hg, and 251 (2.9%) were receiving antihypertensive medications. The distributions of SBP and diastolic BP (DBP) at donation are shown in Figure 2.

### General Characteristics of Donors with Hypertension

Hypertensive donors (*n* = 904) were older (48 vs. 38 years), more likely to be men, less likely to be related to the recipient, and more likely to have a college education (Table 1). Hypertensive donors had a higher weight, BMI, SBP, DBP, and a lower eGFR (83 vs. 89 ml/min/1.73 m^2^). Of the 251 hypertensive donors receiving antihypertensive medications, 154 received 1 agent, 44 received 2, 5 used 3 agents, and in 43 donors this information was missing. Of those treated, 50% were taking an angiotensin-converting enzyme inhibitor or angiotensin-II receptor blocker, 30% were taking diuretic medications, and the remaining received either a calcium channel blocker or a central adrenergic blocker. Hypertensive donors receiving treatment were on average 7 years older than untreated hypertensives, were more likely to be white, were less likely to be related to their recipient, and had a significantly lower SBP and DBP at donation (133 vs. 145 mm Hg for SBP and 80 vs. 84 mm Hg for DBP; Table 2). Importantly, hypertensive donors receiving antihypertensive medications had a 10 ml/min/1.73 m^2^ lower baseline eGFR than hypertensive donors not receiving treatment (*P* < 0.001; Table 2).

**Table 1:** Baseline demographic and clinical characteristics of RELIVE study donors, *n* = 8721

	Normotensive donors, *n* = 7817	Hypertensive donors, *n* = 904	*P* value
Donation to last follow-up, yr, mean ± SD	17.6 ± 10.7	14.3 ± 10.1	<0.001
Age, yr, median (IQR)	38 (30–47)	48 (38–55)	<0.001
<35, *n* (%)	2909 (37.2)	171 (18.9)	
35–50, *n* (%)	3658 (46.8)	358 (39.6)	
>50, *n* (%)	1250 (16.0)	375 (41.5)	
Male, *n* (%)	3373 (43.1)	451 (49.9)	<0.001
Race/ethnicity, *n* (%)			0.08
Non-Hispanic white	6646 (85.0)	766 (84.7)	
Non-Hispanic black	705 (9.0)	99 (11.0)	
Hispanic	150 (1.9)	11 (1.2)	
Asian	71 (0.9)	8 (0.9)	
Other	109 (1.4)	5 (0.6)	
Unknown	136 (1.7)	15 (1.7)	
Related to recipient, *n* (%)	6332 (81.2)	665 (73.9)	<0.001
First-degree relative with hypertension, *n* (%)	2867 (40.2)	405 (48.4)	<0.001
First-degree relative with diabetes, *n* (%)	2848 (39.1)	324 (38.4)	0.71
First-degree relative with kidney disease, *n* (%)	5393 (71.9)	554 (64.0)	<0.001
College or higher education level, *n* (%)	3670 (46.9)	459 (50.8)	0.03
Weight, kg, median (IQR)	74.8 (63.7–86.3)	82.6 (72.1–94.8)	<0.001
BMI, kg/m^2^, median (IQR)	25.5 (22.7–29.0)	28.0 (25.0–31.1)	<0.001
Fasting glucose, mg/dl, median (IQR)	92 (85–99)	96 (89–103)	<0.001
SBP, mm Hg, median (IQR)	120 (112–127)	143 (140–148)	<0.001
DBP, mm Hg, median (IQR)	73 (68–79)	83 (77–88)	<0.001
One artery in nondonated kidney, *n* (%)	4868 (64)	591 (67)	0.06
Left kidney removed, *n* (%)	5508 (71)	658 (73)	0.16
Creatinine, mg/dl, median (IQR)	0.9 (0.8–1.1)	1.0 (0.8–1.1)	<0.001
eGFR, ml/min/1.73 m^2^, median (IQR)	89 (77–103)	83 (71–96)	<0.001

BMI, body mass index; BP, blood pressure; eGFR, estimated glomerular filtration rate; IQR, interquartile range; SD, standard deviation.

**Table 2:** Characteristics of donors with hypertension

	SBP ≥140/90 mm Hg, *n* = 653	Taking antihypertensive medication, *n* = 251	*P* value
Donation to last follow-up, yr, mean ± SD	15.4 ± 10.1	11.7 ± 9.5	<0.001
Age, yr, median (IQR)	45 (35–54)	52 (45–59)	<0.001
<35, *n* (%)	159 (24.3)	12 (4.8)	
35–50, *n* (%)	258 (39.5)	100 (39.8)	
>50, *n* (%)	236 (36.1)	139 (55.4)	
Male, *n* (%)	342 (52.4)	109 (43.4)	0.02
Race/ethnicity, *n* (%)			<0.001
Non-Hispanic white	537 (82.2)	229 (91.2)	
Non-Hispanic black	89 (13.6)	10 (4.0)	
Hispanic	10 (1.5)	1 (0.4)	
Asian	5 (0.8)	3 (1.2)	
Other	2 (0.3)	3 (1.2)	
Unknown	10 (1.5)	5 (2.0)	
Related to recipient, *n* (%)	502 (77.2)	163 (65.2)	<0.001
First-degree relative with hypertension, *n* (%)	249 (41.8)	156 (64.5)	<0.001
First-degree relative with diabetes, *n* (%)	230 (38.1)	94 (39.0)	0.82
First-degree relative with kidney disease, *n* (%)	408 (65.8)	146 (59.6)	0.09
First-degree relative with heart disease, *n* (%)	210 (35.3)	125 (52.3)	<0.001
College or higher education level, *n* (%)	315 (48.2)	144 (57.4)	0.01
Weight, kg, median (IQR)	82.0 (72.0–93.8)	84.6 (73.0–95.7)	0.11
BMI, kg/m^2^, median (IQR)	27.7 (24.8–30.8)	28.9 (26.3–32.3)	<0.001
Fasting glucose, mg/dl, median (IQR)	95 (88–102)	97 (91–104)	0.01
SBP, mm Hg, median (IQR)	145 (142–149)	133 (124–141)	<0.001
DBP, mm Hg, median (IQR)	84 (78–89)	80 (74–85)	<0.001
One artery in nondonated kidney, *n* (%)	428 (68)	163 (66)	0.62
Left kidney removed, *n* (%)	473 (73)	185 (75)	0.60
Creatinine, mg/dl, median (IQR)	1.0 (0.8–1.1)	1.0 (0.9–1.1)	0.44
eGFR, ml/min/1.73 m^2^, median (IQR)	85 (73–98)	76 (67–88)	<0.001

BMI, body mass index; DBP, diastolic blood pressure; eGFR, estimated glomerular filtration rate; IQR, interquartile range; SBP, systolic blood pressure; SD, standard deviation.

**Figure 3:**
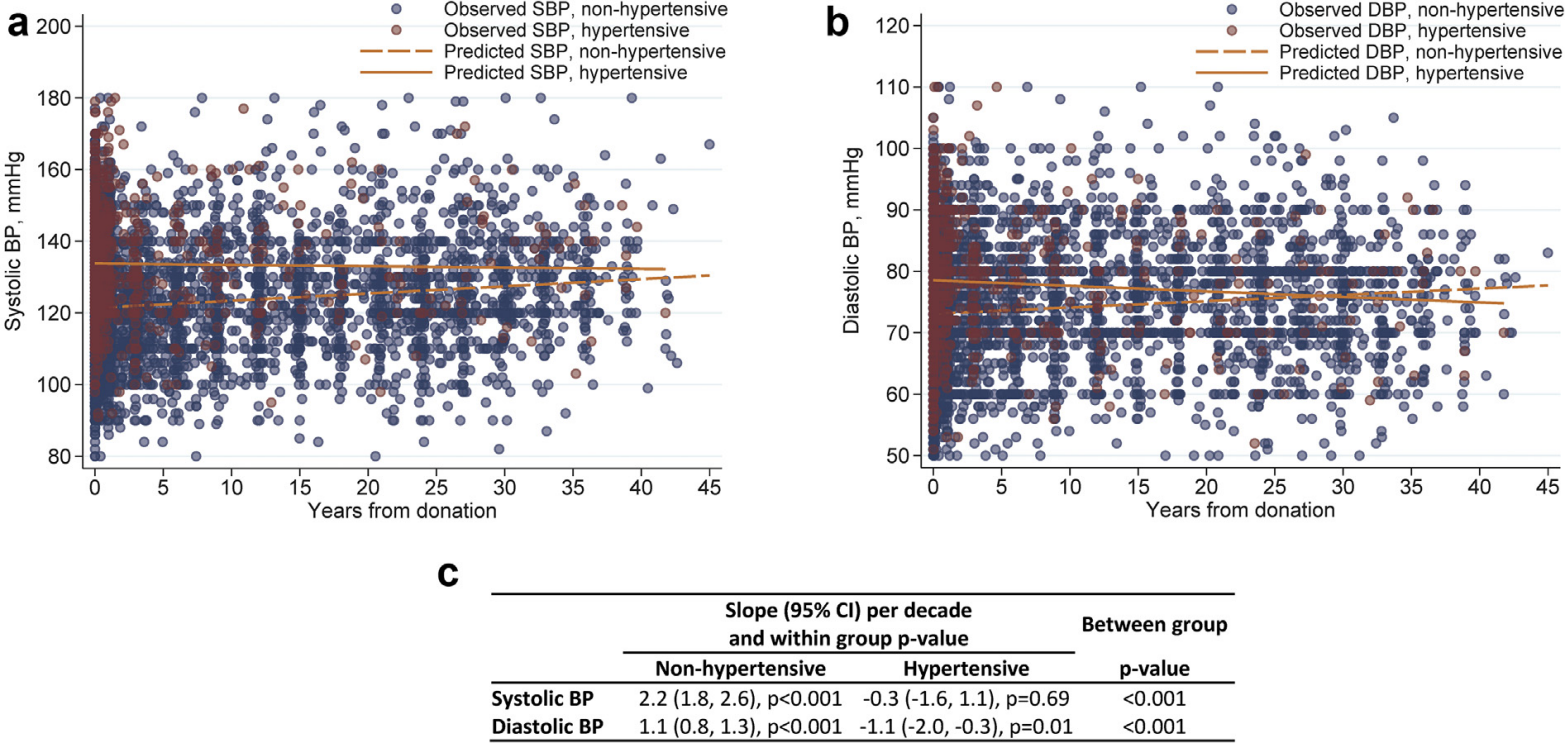
Observed and predicted postdonation blood pressure (BP). (a) Systolic blood pressure (SBP). (b) Diastolic blood pressure (DBP). (c) Slope (95% confidence interval [CI]) of systolic BP and diatolic BP per decade.

We compared the profile of the observed and predicted SBP and DBP in hypertensive and nonhypertensive donors (Figure 3). Observed values of SBP and DBP were obtained from the actual donors SBP and DBP measurements. Predicted SBP and DBP values were obtained using regression line estimates and compared between hypertensive and nonhypertensive donors using linear regression. SBP rose by 2.2 mm Hg per decade (95% confidence interval [CI] 1.8–2.6) in nonhypertensive donors versus −0.3 mm Hg per decade (95% CI −1.6 to 1.1) in hypertensive donors. The difference in slope was significantly different (*P* < 0.001). DBP rose by 1.1 mm Hg per decade (95% CI 0.8–1.3) in nonhypertensive donors versus −1.1 mm Hg per decade (95% CI −2.0 to 0.3) in hypertensive donors (*P* < 0.001).

### Postdonation Hypertension Development

An additional 2319 donors developed hypertension 5.1 ± 9.2 years after donation (Table 3). Donors with postdonation hypertension were younger at donation (40 vs. 48 years of age), were less likely to be related to the recipient, had a higher baseline eGFR, a lower BMI, and were less likely to have a first-degree relative with hypertension. The development of postdonation hypertension was associated with older age (adjusted hazard ratio [aHR] 1.02 [95% CI 1.02–1.03]), male gender (aHR 1.31 [95% CI 1.19–1.44]), BMI (aHR 1.06 [95% CI 1.05–1.08]), and fasting plasma glucose (aHR 1.01 [95% CI 1.006–1.01]; *P* < 0.05 for all).

**Table 3:** Characteristics of donors with predonation and postdonation hypertension

	Predonation hypertension, *n* = 904	Postdonation hypertension, *n* = 2319	*P* value
Donation to last follow-up, yr, mean ± SD	14.3 ± 10.1	21.2 ± 11.1	<0.001
Age, yr, median (IQR)	48 (38–55)	40 (31–48)	<0.001
<35, *n* (%)	171 (18.9)	774 (33.4)	
35–50, *n* (%)	358 (39.6)	1094 (47.2)	
>50, *n* (%)	375 (41.5)	451 (19.4)	
Male, *n* (%)	451 (49.9)	1176 (50.7)	0.67
Race/ethnicity, *n* (%)			0.10
Non-Hispanic white	766 (84.7)	1990 (85.8)	
Non-Hispanic black	99 (11.0)	214 (9.2)	
Hispanic	11 (1.2)	30 (1.3)	
Asian	8 (0.9)	12 (0.5)	
Other	5 (0.6)	38 (1.6)	
Unknown	15 (1.7)	35 (1.5)	
Related to recipient, *n* (%)	665 (73.9)	2002 (86.5)	<0.001
First-degree relative with hypertension, *n* (%)	405 (48.4)	838 (39.4)	<0.001
First-degree relative with diabetes, *n* (%)	324 (38.4)	884 (40.5)	0.28
First-degree relative with kidney disease, *n* (%)	554 (64.0)	1747 (77.7)	<0.001
First-degree relative with heart disease, *n* (%)	335 (40.2)	661 (31.2)	<0.001
College or higher education level, *n* (%)	459 (50.8)	1000 (43.1)	<0.001
Weight, kg, median (IQR)	82.6 (72.1–94.8)	77.9 (66.4–89.9)	<0.001
BMI, kg/m^2^, median (IQR)	28.0 (25.0–31.1)	26.3 (23.4–29.6)	<0.001
Fasting glucose, mg/dl, median (IQR)	96 (89–103)	93 (86–101)	<0.001
SBP, mm Hg, median (IQR)	143 (140–148)	124 (117–130)	<0.001
DBP, mm Hg, median (IQR)	83 (77–88)	76 (71–81)	<0.001
One artery in nondonated kidney, *n* (%)	658 (73)	1636 (71)	0.19
Left kidney removed, *n* (%)	591 (67)	1463 (65)	0.24
Creatinine, mg/dl, median (IQR)	1.0 (0.8–1.1)	1.0 (0.8–1.1)	0.31
eGFR, ml/min/1.73 m^2^, median (IQR)	83 (71–96)	87 (76–101)	<0.001

BMI, body mass index; DBP, diastolic blood pressure; eGFR, estimated glomerular filtration rate; IQR, interquartile range; SBP, systolic blood pressure; SD, standard deviation.

### Outcomes of Interest at Last Follow-Up

After 17.6 ± 10.7 years from donation to last follow-up in 2010 to 2012 in donors without hypertension and 14.3 ± 10.1 years for donors with hypertension, a similar proportion were alive (4.7% vs. 6.0%, *P* = 0.09), had CVD (12.7% vs. 13.9%, *P* = 0.31), and had diabetes (7.1% vs. 8.2%, *P* = 0.26; Table 4). Hypertensive donors were more likely to have proteinuria (17.8% vs. 13.4%, *P* < 0.001) and more likely to have an eGFR < 60 and < 45 ml/min/1.73 m^2^. However, the occurrence of eGFR < 30 ml/min/1.73 m^2^ or ESKD was similar in donors with and without hypertension (Table 4).

**Table 4:** Outcomes of donors by hypertension status at last follow up in 2010 to 2012, n (%)

Outcome	Donors with available data, *n*	*n* (%)	Normotensive donors, *n* = 7817	Hypertensive donors, *n* = 904	*P* value
Donation to last follow-up, yr			17.6 ± 10.7	14.3 ± 10.1	<0.0001
Mortality	8721	422 (4.8)	368 (4.7)	54 (6.0)	0.09
Cardiovascular disease	8706	1118 (12.8)	993 (12.7)	125 (13.9)	0.31
Diabetes	7985	576 (7.2)	507 (7.1)	69 (8.2)	0.26
Proteinuria	7789	1077 (13.8)	930 (13.4)	147 (17.8)	<0.001
eGFR, ml/min/1.73 m^2^					
<60	8469	4718 (55.7)	4117 (54.3)	601 (67.8)	<0.001
<45	8469	1023 (12.1)	857 (11.3)	166 (18.7)	<0.001
<30	8469	60 (0.7)	50 (0.7)	10 (1.1)	0.12
eGFR <30 or ESKD	8658	86 (1.0)	72 (0.9)	14 (1.6)	0.07
ESKD	8116	44 (0.5)	39 (0.5)	5 (0.6)	0.85

eGFR, estimated glomerular filtration rate; ESKD, end-stage kidney disease.

Forty-four donors developed ESKD 19.2 ±10.3 years after donation; 39 occurred in normotensive donors (0.5%) and 5 occurred in hypertensive donors (0.6%). The development of ESKD in hypertensive donors was associated with BMI at donation and having a first-degree relative with hypertension. All 5 ESKD cases in hypertensive donors occurred in non-Hispanic whites, 2 were women, 4 were related to their recipient, 2 had a BMI > 30 kg/m^2^ at donation, and none developed diabetes after donation. One ESKD case occurred in 171 hypertensive donors who were < 35 years of age at donation, 3 occurred in 358 donors 35 to 50 years of age at donation, and 1 developed in 375 donors > 50 years of age at donation. The incidence of ESKD in years 0 to 10, 11 to 20, 21 to 30, and > 30 years after donation is shown in Table 5. The overall ESKD incidence rate was similar in hypertensive and nonhypertensive donors (6.6 [95% CI 4.8–9] vs. 10.9 [95% CI 4.5–75.2]) per 10,000 donor-years (*P* = 0.3). No cases of ESKD occurred in hypertensive donors in the first 10 years, and the incidence after 30 years was 51.8 (95% CI 27–99.6) per 10,000 donor-years in normotensive donors versus 61.5 (95% CI 8.7–436.6) in hypertensive donors (*P* = 0.30).

**Table 5:** ESKD and ESKD or eGFR <30 ml/min/1.73 m^2^ incidence rate (95% CI) per 10,000 donor-years

Outcome	Years				Overall	*P* value
	0–10	11–20	21–30	>30		
ESKD, *n* = 44						
Normotensive donors, *n* = 7265	2.2 (1.0–4.5)	7.6 (4.4–13.0)	12.0 (6.5–22.3)	51.8 (27.0–99.6)	6.6 (4.8–9.0)	0.30
Hypertensive donors, *n* = 851	—	—	—	61.5 (8.7–436.6)	10.9 (4.5–26.1)	
eGFR <30 ml/min/1.73 m^2^ or ESKD, *n* = 86						
Normotensive donors, *n* = 7757	13.7 (9.4–19.9)	12.7 (7.5–21.5)	31.8 (19.8–51.2)	137.6 (81.5–232.3)	19.4 (15.4–24.5)	<0.001
Hypertensive donors, *n* = 901	21.1 (7.9–56.1)	39.9 (12.9–123.9)	101.9 (38.2–271.4)	293.3 (94.6–909.4)	44.5 (26.4–75.2)	

CI, confidence interval; eGFR, estimated glomerular filtration rate; ESKD, end-stage kidney disease.

The composite of eGFR < 30 ml/min/1.73 m^2^ or ESKD occurred in 86 donors; 72 (0.9%) in normotensive donors and 14 (1.6%) in hypertensive donors. The overall rate was 19.4 per 10,000 donors-year (95% CI 15.4–24.5) in normotensive donors versus 44.5 (95% CI 26.4–75.2) in hypertensive donors (*P* < 0.001). The development of this composite in hypertensive donors was associated with age, BMI at evaluation, having a first-degree relative with hypertension, and donation year.

**Figure 4:**
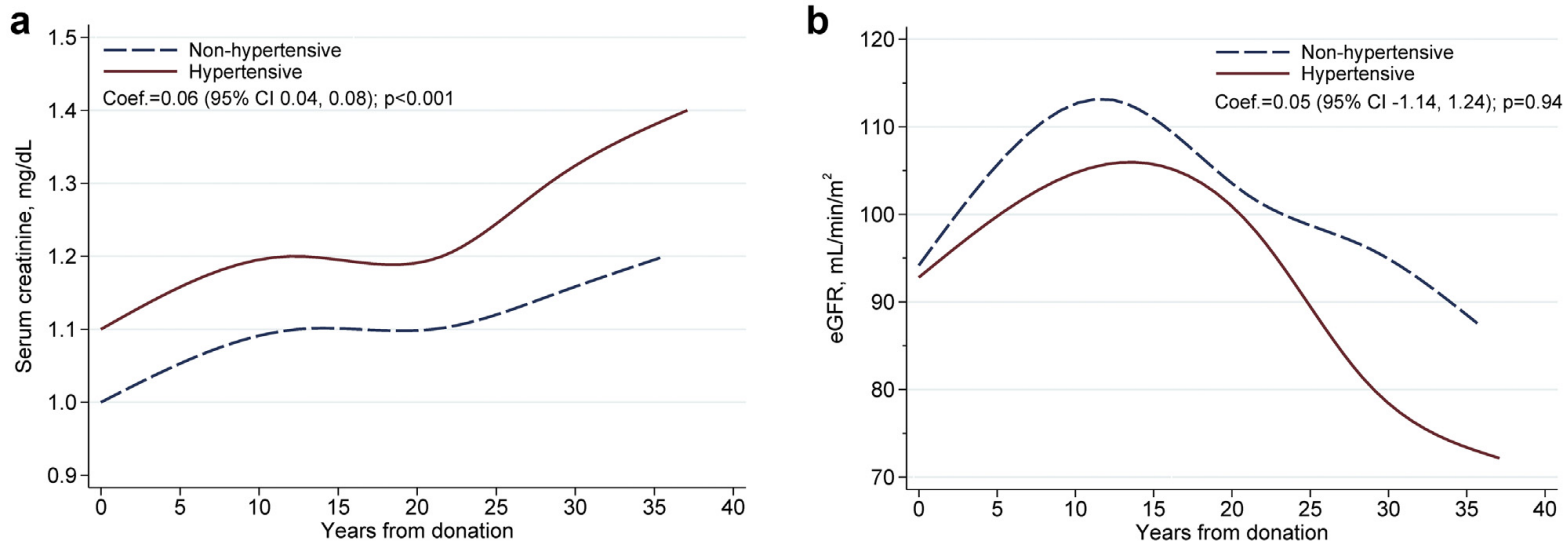
Trajectories of serum creatinine and estimated glomerular filtration rate (eGFR) in hypertensive and nonhypertensive donors. (a) Serum creatinine. (b) eGFR. CI, confidence interval.

Postdonation serum creatinine measurements were available in 99.7% of donors, and 70% had multiple postdonation measurements (4 ± 2.8 measurements/donor), allowing the construction of eGFR trajectory in normotensive and hypertensive donors over time (Figure 4). Hypertensive donors had a significantly higher serum creatinine level over time, but eGFR trajectory was comparable in donors with and without hypertension (coefficient = 0.05 [95% CI −1.14 to 1.24], *P* = 0.94).

### Multivariable Risks of Mortality, CVD, and ESKD

**Figure 5:**
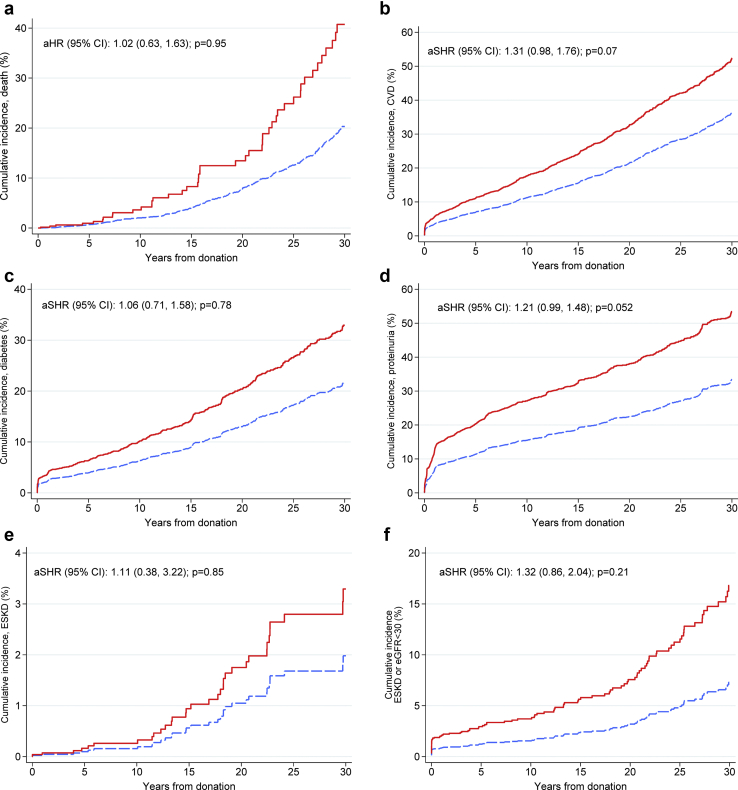
Cumulative incidence of major outcomes. (a) Mortality. (b) Cardiovascular disease (CVD). (c) Diabetes. (d) Proteinuria. (e) End-stage kidney disease (ESKD). (f) Estimated glomerular filtration rate (eGFR) <30 ml/min/1.73m^2^ or end-stage renal disease (ESRD). The red line indicates hypertensive donors and the dashed blue line indicates nonhypertensive donors. aHR, adjusted hazard ratio; aSHR, adjusted subdistribution hazard ratio (obtained from the competing risk analysis); CI, confidence interval.

After adjustment for baseline laboratory values, demographic factors, and the development of diabetes and hypertension after donation, we found that hypertensive donors were not more likely to die, develop cardiovascular disease, develop proteinuria, have a reduced eGFR, or have ESKD. The aHR for ESKD was 1.14 (95% CI 0.62–2.12, *P* = 0.67; Table 6). Similarly, hypertensive donors were not more likely to develop the composite of eGFR < 30 ml/min/1.73 m^2^ or ESKD. Cox regression models run on the multiple imputation dataset yielded similar results with the exception being the CVD outcome, which was significantly associated with hypertension (aHR 1.34 [95% CI 1.05–1.51], *P* = 0.02). The aHR for CVD in the nonimputed model was 1.27 (95% CI 0.96–1.68, *P* = 0.09). Competing risk analysis found no statistically significant differences in the cumulative incidence for any of the outcomes studied between non-hypertensive and hypertensive outcomes (Figure 5).

**Table 6:** Multivariable risk of death, diabetes, CVD, and renal outcomes: Cox regression analysis

	Years from donation to event, mean ± SD	Nonhypertensive donors, *n* (%)	Hypertensive donors, *n* (%)	Complete data		Imputed data	
				aHR (95% CI)	*P* value	aHR (95% CI)	*P* value
Death, *n* = 422/8721	20.7 ± 10.3	368 (4.7)	54 (6.0)	1.02 (0.63–1.63)	0.95	1.28 (0.88–1.87)	0.19
CVD, *n* = 1118/8706	11.6 ± 11.0	993 (12.7)	125 (13.9)	1.27 (0.96–1.68)	0.09	1.34 (1.05–1.72)	0.02
Diabetes, *n* = 576/7985	8.1 ± 10.6	507 (7.1)	69 (8.2)	1.07 (0.72–1.59)	0.74	1.04 (0.72–1.51)	0.83
Proteinuria, *n* = 1077/7789	8.0 ± 10.2	930 (13.4)	147 (17.8)	1.19 (0.97–1.45)	0.09	1.20 (0.99–1.45)	0.06
eGFR, ml/min/1.73 m^2^							
<60, *n* = 4718/8469	3.8 ± 8.3	4117 (54.3)	601 (67.8)	1.02 (0.93–1.12)	0.67	1.01 (0.92–1.10)	0.84
<45, *n* = 1023/8469	5.4 ± 10.5	857 (11.3)	166 (18.7)	1.09 (0.90–1.33)	0.35	1.07 (0.89–1.28)	0.49
<30, *n* = 60/8469	19.3 ± 12.8	50 (0.7)	10 (1.1)	1.28 (0.61–2.67)	0.51	1.19 (0.57–2.46)	0.65
ESKD, *n* = 44/8116	19.2 ± 10.3	39 (0.5)	5 (0.6)	1.14 (0.62–2.12)	0.67	1.01 (0.35–2.88)	0.99
eGFR <30 or ESKD, *n* = 86/8658	16.3 ± 13.1	72 (0.9)	14 (1.6)	1.25 (0.68–2.30)	0.48	1.08 (0.81–1.46)	0.59

aHR, adjusted hazard ratio; CI, confidence interval; CVD, cardiovascular disease; eGFR, estimated glomerular filtration rate; ESKD, end-stage kidney disease; SD, standard deviation.

Multivariable Cox proportional hazard models included systolic blood pressure, donor age, gender, body mass index, fasting glucose, eGFR, smoking, hyperlipidemia, and time-varying variables (development of hypertension and diabetes). eGFR <30, ESKD, and eGFR <30 or ESKD models were adjusted for age, gender, body mass index, and eGFR given the small number of events.

Lastly, 1465 donors fulfilled the new definition of hypertension, and the analyses using the newly introduced definition of hypertension (≥130/80 mm Hg) are presented in their entirety as a supplement (Supplementary Tables S1 through S6 and Supplementary Figures S1 through S5). Donors who fulfilled the more recent definition of hypertension had comparable renal outcomes to nonhypertensive donors but were more likely to die (aHR 1.29 [95% CI 1.0–1.67], *P* = 0.051), were more likely to develop CVD (aHR 1.36 [95% CI 1.15–1.61], *P* < 0.001), and were more likely to develop diabetes (aHR 1.41 [95% CI 1.08–1.83], *P* = 0.01).

In conclusion, our results suggest that hypertensive donors, compared with nonhypertensive donors, are not at increased risk for reduced eGFR, proteinuria, or ESKD. Hypertensive donors by previous definition were not more likely to die or develop CVD, either. Donors fulfilling the new definition, however, were more likely to develop CVD and diabetes. These results also show that roughly a third of kidney donors developed hypertension after donation.

The focus of this analysis was to address the long-term outcomes of donors fulfilling the old hypertension definition at donation because this was the prevalent definition during most of the study period. In total, 5 white donors (3 receiving antihypertensive medications at donation and 2 with BP ≥ 140/90 mm Hg) developed ESKD 19.2 ± 10.3 years after donation. To gain a better perspective regarding the incidence of ESKD in hypertensive donors, were compared it to ESKD incidence in 2 external groups from the published literature. In a Kaiser Permanente study, enrollees with an eGFR ≥ 60 ml/min/1.73 m^2^, no proteinuria, no hematuria, and no diabetes were followed for ESKD development.^18^ The overall incidence of ESKD in normotensive and hypertensive enrollees were 8.9 and 32 per 100,000 person-years compared with 6.2 and 12.4 per 100,000 person-years in kidney donors in the RELIVE study cohort. We also compared the observed ESKD rate in RELIVE hypertensive donors to the rate of ESKD attributed to hypertensive nephrosclerosis in the larger U.S. kidney donors reported by Anjum *et al.*^4^ The 10- and 25-year incidence in hypertensive RELIVE donors were 0.2 per 10,000 donors and 3.1 per 10,000 donors compared with 0.6 per 10,000 donors and 2.9 per 10,000 donors in the larger U.S. kidney donor population. These 2 indirect comparisons provide some assurance that the risk of ESKD in hypertensive donors may not be increased, and its rarity provides a possible need to rethink the strength of the link between hypertension and CKD and perhaps the soundness of declining many individuals from donating because they have hypertension. The firmly held belief that hypertension is associated with ESKD development has come from large epidemiologic studies such as the Multiple Risk Factors Intervention Trial, studies in veterans, and from a large cohort of Japanese individuals who were followed prospectively in a mass screening program.^19^, ^20^, ^21^ Collectively, however, these studies lacked objective assessment of kidney function (urinary protein and serum creatinine measurements) in a substantial number of participants, making it difficult to rule out the presence of an underlying intrinsic kidney disease at the beginning of follow-up. To clarify this issue, Hsu *et al.*^18^ studied 316,676 adult Kaiser Permanente members who had an eGFR >60 ml/minute/1.73 m^2^ and no proteinuria or hematuria at cohort entry who were followed for ESKD development. There was a graded association between BP level and ESKD, and the overall incidence rate of ESKD was 14.3 per 10,000 person-years. Interestingly, the ESKD rate in hypertensive RELIVE donors, as noted above, was actually lower than that reported by Hsu *et al.*,^18^ which is not surprising considering how much healthier donors are compared with individuals from the general population. This low likelihood of developing ESKD related to hypertension was also recently reported by Grams *et al.*^22^ The lifetime risk of ESKD attributable to hypertension in multiple contemporary cohorts of subjects having a low risk for CKD (akin to but not as healthy as kidney donors) almost never exceeds 1% in white patients but approaches 3% in African American (AA) patients. The latter is important because hypertension may indeed be causally related to CKD development in AA patients, particularly those who harbor an adverse *APOL1* polymorphism.^23^ Of note, there were 99 AA donors (range 30–57 years of age) with hypertension in this cohort and none developed ESKD after 13.5 ± 8.3 years of follow-up. Previous studies showing that both AA and Hispanic donors have higher rates of hypertension early after donation makes these 2 groups particularly important for future high-quality investigations and special considerations regarding their candidacy for donation.^24^

ESKD is fortunately a rare event after donation, and therefore most if not all studies addressing this important subject lack the statistical power to make overreaching conclusions regarding ESKD development. The current analysis is no exception. The ability to show that more common intermediate renal outcomes, namely proteinuria and eGFR trajectory, were comparable in hypertensive and nonhypertensive donors remedies the issue of low power, albeit only partially. We certainly hope that we will always be underpowered in our studies of ESKD in kidney donors because that would be reflective of diligence and protection donors need and deserve.

While the literature is not consistent regarding whether hypertension is more common in kidney donors, Holscher *et al.*^5^ found that kidney donation was associated with a 19% higher risk of self-reported hypertension when compared with healthy nondonor control subjects drawn from the Atherosclerosis Risk in Communities study and the Coronary Artery Risk Development in Young Adults study. In the current analysis, SBP rose by 2.2 mm Hg per decade in donors without hypertension at donation, which is highly comparable to our previously described rate of 2.9 mm Hg per decade in a longitudinal study of 4296 kidney donors who donated between 1963 and 2014.^25^ Of note, the typical SBP rise per decade in the general population is estimated at 7 mm Hg.^26^

Almost half of U.S. transplant centers exclude donor candidates taking any antihypertensive medications, and 41% exclude donors taking >1 antihypertensive agent.^7^ Based on the data we provide here, we propose that hypertensive donors can perhaps be considered for donation more liberally as long as their BP is well controlled (as confirmed by ambulatory BP monitoring), they have no proteinuria and they have no end organ damage (no left ventricular hypertrophy or hypertensive retinopathy). Restricting the acceptance of hypertensive donors to those above a certain age (usually 50 years of age) is common. In this cohort, 60% of hypertensive donors were < 50 years of age, 20% were < 35 years of age, and 4 of 5 of the ESKD cases occurred in patients < 50 years of age at donation. Therefore, this arbitrarily chosen age of 50 years may not be unreasonable. Certainly, younger age in a hypertensive donor of AA or Hispanic ethnicity may carry a risk for ESKD that would be considered prohibitive by many and certainly require more extensive informed consent. While analyses using the new definition of hypertension yielded similar results for renal outcomes, donors with BP ≥ 130/80 mm Hg or who were receiving treatments were more likely to die and have CVD. We suspect his is largely because of the larger number of events observed in these donors. The magnitude of the association with mortality and CVD appears to be in line with what is seen in people with 2 kidneys.^11^

These analyses have strengths. The population studied spans 50 years of kidney donation, is ethnically diverse, and donors had ascertainable intermediate renal outcomes, such as reduced GFR, serial serum creatinine availability, proteinuria assessment, and CVD, which are not captured in national donor databases. There are limitations, however. Donors included in this analysis come from 3 major U.S. transplant centers with a long-standing tradition in live kidney donation and while ethnically diverse, the proportion of non-Hispanic white donors in the RELIVE study was significantly higher than what is observed in the larger U.S. donor pool, which is approximately 70%. Moreover, the proportion of Hispanic and Asian donors were less than what is observed nationally. The RELIVE study public dataset does not have the cause of ESKD in donors and it would also have been ideal to know how kidneys from hypertensive donors fared in the recipients. Importantly, there probably was no standardization of how BP measurements were carried out at the 3 centers, and many donors labeled as hypertensive may have simply had white coat hypertension. To at least partially address the latter, the average of the 3 lowest BP measurements were used as baseline. Many normotensive donors, on the other hand, may have had masked hypertension, which is also not captured. ESKD was not ascertained by linkage to the Organ Procurement and Transplantation Network or the U.S. Renal Data System, which is why we focused on the more common intermediate renal outcomes and eGFR trajectory analyses. In all, however, kidney donor studies addressing ESKD suffer from low statistical power because of the low event rate of its occurrence. The eGFR trajectory analysis revealed a surprisingly high eGFR in all donors. We are not certain we have an explanation for this finding. Possibilities include the known poor performance of eGFR estimating models in those with GFR >60 ml/min and the fact that the serum creatinine assay has certainly changed over the almost 5 decades of the RELIVE study. To make sure that there was no ascertainment bias obtaining serum creatinine measurements more often in donors with hypertension, we found that both hypertensive and nonhypertensive donors had a similar average of 3 measurements after donation. Therefore, the latter is less likely to explain this observation.

In all, these data show that predominantly non-Hispanic white hypertensive donors do not have an increased risk of ESKD compared with normotensive kidney donors. Importantly, the more common events of eGFR change after donation and proteinuria development were also similar between hypertensive and normotensive donors. We believe most donor candidates with hypertension, particularly white donors, can be considered for donation provided that subtle renal disease is ruled out and the hypertensive candidate is not at a magnified risk for future CVD from hypertension presence on the background of other risk factors.

## Disclosure

All the authors declared no competing interests.

## Supplementary Material

Supplementary File (PDF)

**Table S1.** Baseline demographic and clinical characteristics of RELIVE study donors (*n* = 8721).

**Table S2.** Characteristics of donors with hypertension.

**Table S3.** Characteristics of donors with predonation and postdonation hypertension.

**Table S4.** Outcomes of donors by hypertension status at donation at last follow-up (in 2010–2012).

**Table S5.** ESKD and ESKD or eGFR <30 ml/min/1.73 m^2^ incidence rate, per 10,000 donor-years.

**Table S6.** Multivariable risk of death, diabetes, hypertension, cardiovascular disease (CVD) and renal outcomes - Cox regression analysis in all donors.

**Figure S1.** Flowchart of the study population.

**Figure S2.** Observed and predicted postdonation blood pressure.

**Figure S3.** Trajectories of serum creatinine and eGFR in hypertensive and nonhypertensive donors.

**Figure S4.** Cumulative incidence of major outcomes. (a) Mortality. (b) CVD. (c) Diabetes. (d) Proteinuria. (e) ESKD. (f) eGFR <30 ml/min/1.73 m^2^, or ESRD.
