# Supplementary material for: Outcomes of Hypertensive Kidney Donors Using Current and Past Hypertension Definitions
Source: Kidney Int Rep. 2021 Mar 3;6(5):1242–53. doi: 10.1016/j.ekir.2021.02.034 (PMC8116910; doi:10.1016/j.ekir.2021.02.034)
Supplement: Supplementary File (PDF) [file mmc1.pdf]

Table S1. Baseline demographic and clinical characteristics of RELIVE Study donors (n-8721)

	Normotensive donors (*n*=6352)	Hypertensive donors (*n*=2369)	p-value
Donation to last follow-up(year)	18.1 ±10.8	15.1 ±10.2	<0.001
Age (year)	38 (30, 46)	44 (34, 52)	<0.001
<35	2453 (38.6)	627 (26.5)	
35 – 50	2995 (47.2)	1021 (43.1)	
>50	904 (14.2)	721 (30.4)	
Male	2507 (39.5)	1317 (55.6)	<0.001
Race/ethnicity			
Non-Hispanic White	5430 (85.5)	1982 (83.7)	0.003
Non-Hispanic Black	538 (8.5)	266 (11.2)	
Hispanic	121 (1.9)	40 (1.7)	
Asian	59 (0.9)	20 (0.8)	
Other	89 (1.4)	25 (1.1)	
Unknown	115 (1.8)	36 (1.5)	
Related to recipient	5180 (81.8)	1817 (76.9)	<0.001
1st degree relative with hypertension	2311 (36.4)	961 (40.6)	<0.001
1st degree relative with diabetes	2342 (36.9)	830 (35.0)	0.11
1st degree relative with kidney disease	4419 (69.6)	1528 (64.5)	<0.001
Weight (kg)	72.9 (62.5, 84.4)	82.1 (71.4, 93.8)	<0.001
BMI (kg/m^2)	25.1 (22.4, 28.5)	27.7 (24.6, 30.8)	<0.001
Fasting glucose (mg/dL)	91 (85, 98)	95 (87, 102)	<0.001
Systolic BP (mmHg)	117 (111, 123)	136 (132, 141)	<0.001
Diastolic BP (mmHg)	72 (67, 77)	80 (75, 85)	<0.001
One artery in non-donated kidney	3956 (64)	1503 (65)	0.28
Left kidney removed	4456 (71)	1710 (73)	0.09
Creatinine (mg/dL)	0.9 (0.8, 1.1)	1.0 (0.9, 1.1)	<0.001
eGFR (ml/min/1.73 m^2)	89 (77, 103)	85 (74, 98)	<0.001

Values are in frequency (%), median (interquartile range) or mean ±SD; BMI, body mass index; BP, blood pressure; eGFR estimated glomerular filtration rate.

Table S2. Characteristics of donors with hypertension/

	**SBP ≥130/80 mmHg (*n*=2118)**	**On antihypertensives (*n*=251)**	**p-value**
Donation to last follow-up(year)	15.5 ±10.2	11.7 ±9.5	<0.001
Age (year)	42 (33, 51)	52 (45, 59)	<0.001
<35	615 (29.0)	12 (4.8)	
35 – 50	921 (43.5)	100 (39.8)	
>50	582 (27.5)	139 (55.4)	
Male	1208 (57.0)	109 (43.4)	<0.001
Race/ethnicity			
Non-Hispanic White	1753 (82.8)	229 (91.2)	0.002
Non-Hispanic Black	256 (12.1)	10 (4.0)	
Hispanic	39 (1.8)	1 (0.4)	
Asian	22 (1.0)	3 (1.2)	
Other	17 (0.8)	3 (1.2)	
Unknown	31 (1.5)	5 (2.0)	
Related to recipient	1654 (78.3)	163 (65.2)	<0.001
1st degree relative with hypertension	942 (44.5)	144 (57.4)	<0.001
1st degree relative with diabetes	805 (38.0)	156 (62.2)	<0.001
1st degree relative with kidney disease	736 (34.7)	94 (37.5)	0.40
1st degree relative with heart disease	1382 (65.3)	146 (58.2)	0.027
Weight (kg)	80.3 (75.3, 85.3)	80.0 (74.3, 85.3)	0.12
BMI (kg/m^2)	27.4 (24.5, 30.6)	28.9 (26.3, 32.3)	<0.001
Fasting glucose (mg/dL)	94 (87, 102)	97 (91,104)	<0.001
Systolic BP (mmHg)	136 (132, 141)	133 (124, 141)	<0.001
Diastolic BP (mmHg	80 (75, 85)	80 (74, 85)	0.12
One artery in non-donated kidney	1340 (65)	163 (66)	0.81
Left kidney removed	1525 (73)	185 (75)	0.48
Creatinine (mg/dL)	1.0 (0.9, 1.1)	1.0 (0.9, 1.1)	0.98
eGFR (ml/min/1.73 m^2)	86 (75, 99)	76 (67, 88)	<0.001

Values are in frequency (%), median (interquartile range) or mean ±SD; BMI, body mass index; BP, blood pressure; eGFR estimated glomerular filtration rate.

Table S3. Characteristics of donors with pre-donation and post-donation hypertension

	Pre-donation Hypertension (*n*=2369)	Post-donation Hypertension (*n*=1647)	p-value
Donation to last follow-up(year)	15.1 ±10.2	22.5 ±10.9	<0.001
Age (year)	44 (34, 52)	39 (31, 47)	<0.001
<35	627 (26.5)	585 (35.5)	
35 – 50	1021 (43.1)	794 (48.2)	
>50	721 (30.4)	268 (16.3)	
Male	1317 (55.6)	758 (46.0)	<0.001
Race/ethnicity			
Non-Hispanic White	1982 (83.7)	1422 (86.3)	0.02
Non-Hispanic Black	266 (11.2)	147 (8.9)	
Hispanic	40 (1.7)	19 (1.2)	
Asian	20 (0.8)	7 (0.4)	
Other	25 (1.1)	28 (1.7)	
Unknown	36 (1.5)	24 (1.5)	
Related to recipient	1817 (76.9)	1457 (88.7)	<0.001
1st degree relative with hypertension	1086 (45.8)	720 (43.7)	0.18
1st degree relative with diabetes	961 (40.6)	588 (35.7)	0.002
1st degree relative with kidney disease	830 (35.0)	647 (39.3)	0.01
1st degree relative with heart disease	1528 (64.5)	1281 (77.8)	<0.001
Weight (kg)	80 (75, 85)	74 (70, 79)	<0.001
BMI (kg/m^2)	27.7 (24.6, 30.8)	25.8 (22.9, 29.2)	<0.001
Fasting glucose (mg/dL)	95 (87, 102)	92 (85, 100)	<0.001
Systolic BP (mmHg)	136 (132, 141)	120 (113, 125)	<0.001
Diastolic BP (mmHg)	80 (75, 85)	74 (70, 79)	<0.001
One artery in non-donated kidney	1503 (65)	1038 (65)	0.81
Left kidney removed	1710 (72)	1152 (70)	0.11
Creatinine (mg/dL)	1.0 (0.9, 1.1)	0.9 (0.8, 1.1)	<0.001
eGFR (ml/min/1.73 m^2)	85 (78, 98)	88 (76, 101)	<0.001

Values are in frequency (%), median (interquartile range) or mean ±SD; BMI, body mass index; BP, blood pressure; eGFR estimated glomerular filtration rate.

Table S4. Outcomes of donors by hypertension status at donation at last follow-up (in 2010-2012)

Outcome	Donor with data available	n (%)	Normotensive donors (*n*=6352)	Hypertensive donors (*n*=2369)	p- value
Donation to last follow-up (year)	8721	--	18.1 (±10.8)	15.1 (±10.2)	<0.001
Mortality	8721	422 (4.8)	296 (4.7)	126 (5.3)	<0.20
Cardiovascular disease	8706	1118 (12.8)	785 (12.4)	333 (14.1)	0.03
Diabetes	7985	576 (7.2)	385 (6.6)	191 (8.8)	0.001
Hypertension	8468	2664 (31.5)	1627 (25.6)	1037 (49.0)	<0.001
Proteinuria	7789	1077 (13.8)	760 (13.4)	317 (15.0)	0.08
eGFR <60 ml/min/1.73 m^2	8469	4718 (55.7)	3271 (53.1)	1447 (62.7)	<0.001
eGFR <45 ml/min/1.73 m^2	8469	1023 (12.1)	645 (10.5)	378 (16.4)	<0.001
eGFR <30 ml/min/1.73 m^2	8469	60 (0.7)	36 (0.6)	24 (1.0)	0.03
eGFR<30 or ESKD	8658	86 (1.0)	54 (0.9)	32 (1.4)	0.04
ESKD	8116	44 (0.5)	32 (0.5)	12 (0.5)	1.00

Table S5. ESKD and ESKD or eGFR<30ml/min /1.73 m2 incidence rate, per 10,000 donor-years

Outcome	0-10 years	11-20 years	21-30 years	>30 years	Overall	p-value
**ESKD (*n*=44)**						
Normotensive donors (n=5902)	2.2 (1.0, 4.9)	7.9 (4.4, 13.8)	10.8 (5.4, 21.7)	41.0 (18.4, 91.2)	6.2 (4.4, 8.8)	0.21
Hypertensive donors (n=2214)	1.3 (0.2, 9.2)	17.2 (7.1, 41.2)	13.6 (3.4, 54.4)	92.2 (34.6, 245.7)	9.6 (5.4, 16.9)	
**eGFR <30 or ESKD (*n*=86)**						
Normotensive donors (n=6303)	11.9 (7.7, 18.5)	11.2 (6.2, 20.2)	29.6 (17.6, 50.1)	103.0 (53.6, 197.9)	16.8 (12.9, 21.9)	<0.001
Hypertensive donors (n=2355)	22.4 (12.4, 40.4)	31.8 (14.3, 70.7)	69.4 (33.1, 145.6)	325.5 (162.8, 650.8)	39.7 (28.1, 650.8)	

Table S6. Multivariable risk of death, diabetes, hypertension, cardiovascular disease (CVD) and renal outcomes - Cox regression analysis in all donors

	Donation to event Mean ±SD	Normotensive donors	Hypertensive donors	Complete data		Imputed data	
		n (%)	n (%)	aHR (95% CI)	p-value	aHR (95% CI)	p-value
Death (n=422/8721)	20.7 ±10.3	296 (4.7)	126 (5.3)	1.35 (0.97, 1.88)	0.07	1.29 (1.00, 1.67)	0.051
CVD (n=1118/8706)	11.6 ±11.0	785 (12.4)	333 (14.1)	1.32 (1.08, 1.61)	0.01	1.36 (1.15, 1.61)	<0.001
Diabetes (n=576/7985)	8.1 ±10.6	385 (6.6)	191 (8.8)	1.47 (1.12, 1.93)	0.01	1.41 (1.08, 1.83)	0.01
Proteinuria (n=1077/7789)	8.0 ±10.2	760 (13.4)	317 (15.0)	1.12 (0.97, 1.30)	0.13	1.09 (0.94, 1.26)	0.24
eGFR <60 (n=4718/8469)	3.8 ±8.3	3271 (53.1)	1447 (62.7)	1.02 (0.95, 1.10)	0.56	1.03 (0.96, 1.09)	0.43
eGFR <45 (n=1023/8469)	5.4 ±10.5	645 (10.5)	378 (16.4)	1.01 (0.87, 1.18)	0.89	1.01 (0.87, 1.16)	0.91
eGFR <30 (n=60/8469)	19.3 ±12.8	36 (0.6)	24 (1.0)	1.74 (0.99, 3.05)	0.054	1.64 (0.92, 2.92)	0.09
ESRD (n=44/8116)	19.2 ±10.)	32 (0.5)	12 (0.5)	1.15 (0.56, 2.35)	0.71	1.09 (0.54, 2.19)	0.81
eGFR<30 or ESRD (n=86/8658)	16.3 ±13.1	54 (0.9)	32 (1.4)	1.49 (0.93, 2.38)	0.10	1.43 (0.90, 2.26)	0.13

Supplemental Figure S1. Flowchart of the study population

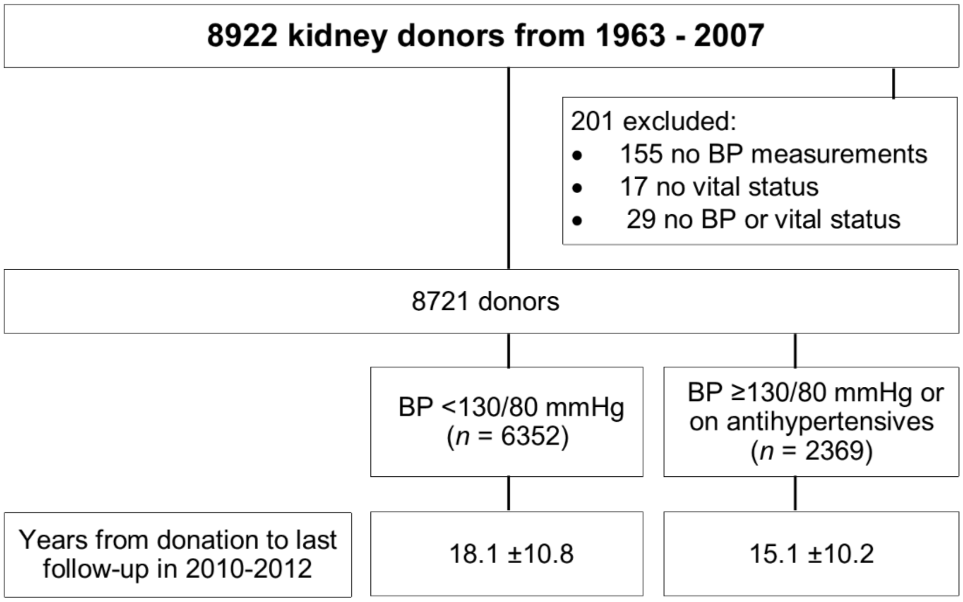

Supplemental Figure S2. Observed and predicted post-donation blood pressure:
A. Systolic blood pressure; B. Diastolic blood pressure; C. Slope (95% CI) of systolic and diastolic blood pressure per decade

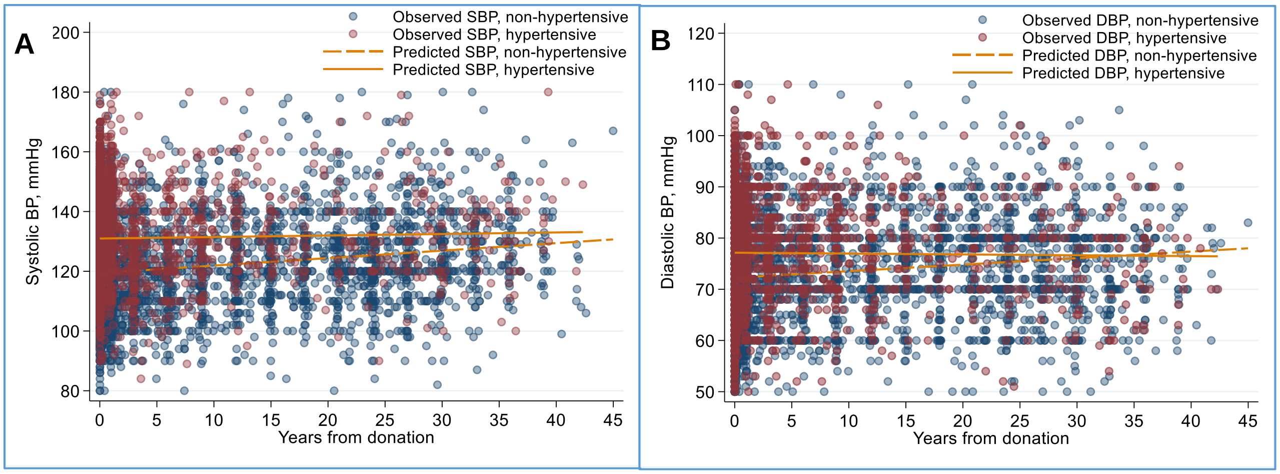

C

	Slope (95% CI) per decade and within group p-value		Between group
	**Non-hypertensive**	**Hypertensive**	**p-value**
**Systolic BP**	2.7 (2.3, 3.1), p<0.001	0.7 (-0.2, 1.5), p=0.12	<0.001
**Diastolic BP**	1.4 (1.1, 1.6), p<0.001	-0.3 (-0.9, 0.2), p=0.23	<0.001

Supplemental Figure S3. Trajectories of serum creatinine and eGFR in hypertensive and non-hypertensive donors

A. Serum creatinine

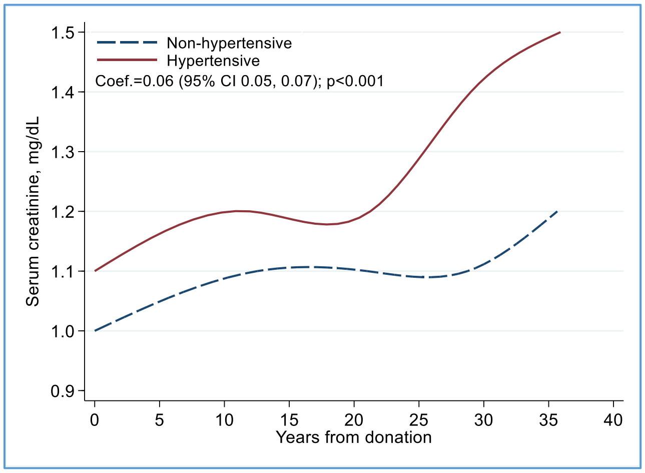

B. eGFR

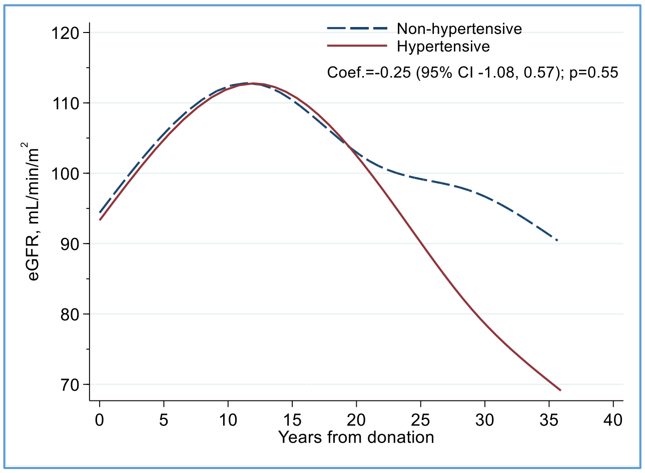

Supplemental Figure S4. Cumulative incidence of major outcomes: A. Mortality; B. CVD; C. Diabetes; D. Proteinuria; E. ESKD; F. eGFR <30 ml/min/1.73m2 or ESRD

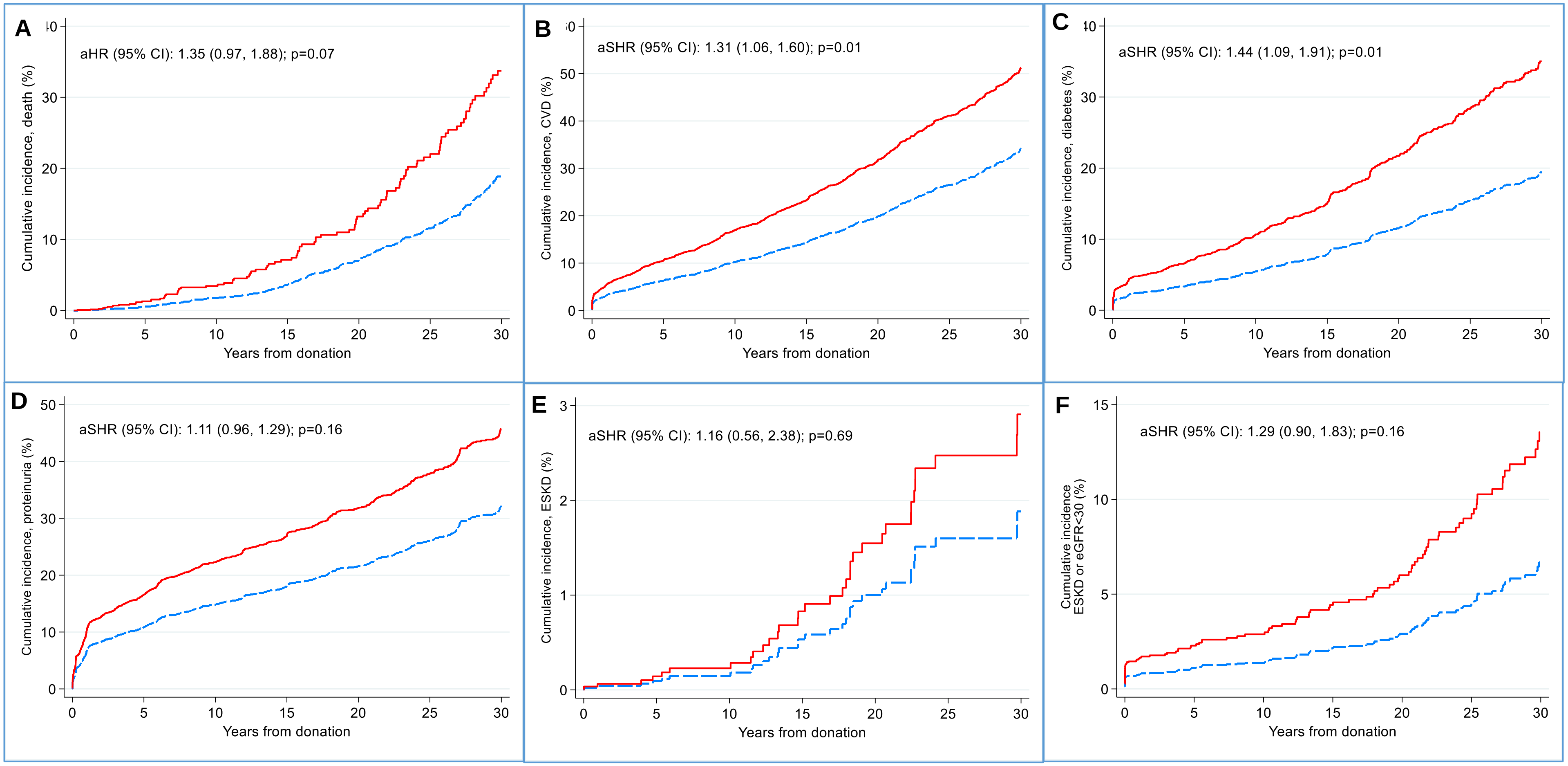

——— Hypertensive donors

---------- Non-hypertensive donors

HR, hazard ratio; SHR, sub-distribution hazard ratio (obtained from the competing risk analysis); CI, confidence interval.

STROBE (Strengthening The Reporting of OBservational Studies in Epidemiology) Checklist

A checklist of items that should be included in reports of observational studies. You must report the page number in your manuscript where you consider each of the items listed in this checklist. If you have not included this information, either revise your manuscript accordingly before submitting or note N/A.

Note: An Explanation and Elaboration article discusses each checklist item and gives methodological background and published examples of transparent reporting. The STROBE checklist is best used in conjunction with this article (freely available on the Web sites of PLoS Medicine at http://www.plosmedicine.org/, Annals of Internal Medicine at http://www.annals.org/, and Epidemiology at http://www.epidem.com/). Information on the STROBE Initiative is available at www.strobe-statement.org.

Section and Item	Item No.	Recommendation	Reported on Page No.
**Title and Abstract**	1	(*a*) Indicate the study's design with a commonly used term in the title or the abstract	1, 2
		(*b*) Provide in the abstract an informative and balanced summary of what was done and what was found	2
**Introduction**			
Background/Rationale	2	Explain the scientific background and rationale for the investigation being reported	3, 4
Objectives	3	State specific objectives, including any prespecified hypotheses	3
**Methods**			
Study Design	4	Present key elements of study design early in the paper	4, 5
Setting	5	Describe the setting, locations, and relevant dates, including periods of recruitment, exposure, follow-up, and data collection	4, 5
Participants	6	(*a*) *Cohort study*—Give the eligibility criteria, and the sources and methods of selection of participants. Describe methods of follow-up	4, 5
		Case-control study—Give the eligibility criteria, and the sources and methods of case ascertainment and control selection. Give the rationale for the choice of cases and controls	NA
		Cross-sectional study—Give the eligibility criteria, and the sources and methods of selection of participants	NA
		(*b*) *Cohort study*—For matched studies, give matching criteria and number of exposed and unexposed	NA
		Case-control study—For matched studies, give matching criteria and the number of controls per case	NA
Variables	7	Clearly define all outcomes, exposures, predictors, potential confounders, and effect modifiers. Give diagnostic criteria, if applicable	5, 6

Section and Item	Item No.	Recommendation	Reported on Page No.
Data Sources/ Measurement	8*	For each variable of interest, give sources of data and details of methods of assessment (measurement). Describe comparability of assessment methods if there is more than one group	5, 6
Bias	9	Describe any efforts to address potential sources of bias	5, 6
Study Size	10	Explain how the study size was arrived at	4
Quantitative Variables	11	Explain how quantitative variables were handled in the analyses. If applicable, describe which groupings were chosen and why	5, 6
Statistical Methods	12	(*a*) Describe all statistical methods, including those used to control for confounding	6
		(*b*) Describe any methods used to examine subgroups and interactions	6
		(*c*) Explain how missing data were addressed	6
		(*d*) *Cohort study*—If applicable, explain how loss to follow-up was addressed	6
		Case-control study—If applicable, explain how matching of cases and controls was addressed	NA
		Cross-sectional study—If applicable, describe analytical methods taking account of sampling strategy	NA
		(*e*) Describe any sensitivity analyses	NA
**Results**			
Participants	13*	(a) Report numbers of individuals at each stage of study—eg numbers potentially eligible, examined for eligibility, confirmed eligible, included in the study, completing follow-up, and analysed	7
		(b) Give reasons for non-participation at each stage	6
		(c) Consider use of a flow diagram	25
Descriptive Data	14*	(a) Give characteristics of study participants (eg demographic, clinical, social) and information on exposures and potential confounders	4-7
		(b) Indicate number of participants with missing data for each variable of interest	7
		(c) *Cohort study*—Summarise follow-up time (eg, average and total amount)	7
Outcome Data	15*	*Cohort study*—Report numbers of outcome events or summary measures over time	7-10
		Case-control study—Report numbers in each exposure category, or summary measures of exposure	NA
		Cross-sectional study—Report numbers of outcome events or summary measures	NA

Section and Item	Item No.	Recommendation	Reported on Page No.
Main Results	16	(*a*) Give unadjusted estimates and, if applicable, confounder-adjusted estimates and their precision (eg, 95% confidence interval). Make clear which confounders were adjusted for and why they were included	7-10
		(*b*) Report category boundaries when continuous variables were categorized	7-10
		(*c*) If relevant, consider translating estimates of relative risk into absolute risk for a meaningful time period	NA
Other Analyses	17	Report other analyses done—eg analyses of subgroups and interactions, and sensitivity analyses	Supplement
**Discussion**			
Key Results	18	Summarise key results with reference to study objectives	10
Limitations	19	Discuss limitations of the study, taking into account sources of potential bias or imprecision. Discuss both direction and magnitude of any potential bias	14
Interpretation	20	Give a cautious overall interpretation of results considering objectives, limitations, multiplicity of analyses, results from similar studies, and other relevant evidence	14, 15
Generalisability	21	Discuss the generalisability (external validity) of the study results	13-15
**Other Information**			
Funding	22	Give the source of funding and the role of the funders for the present study and, if applicable, for the original study on which the present article is based	NA

*Give information separately for cases and controls in case-control studies and, if applicable, for exposed and unexposed groups in cohort and cross-sectional studies.

Once you have completed this checklist, please save a copy and upload it as part of your submission. DO NOT include this checklist as part of the main manuscript document. It must be uploaded as a separate file.
